# Serum brain-derived neurotrophic factor (BDNF) as predictors of childhood neuroblastoma relapse

**DOI:** 10.1186/s12885-023-11159-9

**Published:** 2023-07-17

**Authors:** Xilin Xiong, Meiling Zeng, Xiaomin Peng, Chuchu Feng, Chunmou Li, Wenjun Weng, Yang Li

**Affiliations:** Department of Oncology, Medical Centre of Pediatric, Sun Yat-sen Memorial Hospital, Sun Yat-sen University, No 107 Yanjiang Road, Guangzhou, Guangdong 510120 China

**Keywords:** Neuroblastoma, Relapse, BDNF

## Abstract

**Background:**

Neuroblastoma (NB) is a childhood malignant tumor,50% of high-risk NB children still have recurrence, and the long-term survival rate is very low. NB tumors expressing high levels of BDNF/TrkB are associated with poor survival outcomes.In this study, we show that the trends of serum concentration of BDNF at different growth stages after birth, and explore the relationship with NB replase.

**Methods:**

In experiment 1, 87 subjects were enrolled and divided into four groups, neonates group、 children group、adults group and NB patients. The distribution of serum concentration of BDNF by ELISA. In experiment 2, we studied BDNF in stage 4 NB patients to determine their frequency, correlation with clinical parameters, and prognostic impact.

**Results:**

First, we identified that serum BDNF concentration decreased from the newborn to childhood in healthy subjects, while it was relatively high in children(age > 1 year) with NB. In the second phase our studies showed no significant increase in serum BDNF concentration in these NB patients, with adverse pathologic features, large tumor maximum diameter, and MYCN amplification. After comprehensive treatment, levels of BDNF gradually increased in children with recurrence and decreased in the remission group. High serum BDNF concentration was associated with relapse. Of 21 stage 4 neuroblastoma patients, adopted a comprehensive treatment approach including ATO-basic modified chemotherapy, traditional radiotherapy,stem cell transplatation and immunotherapy. 76% of alive patients having > 3 years follow-up. Conclusion:The aim is to show that BDNF is a predictor of recurrence risk of NB.

## Introduction

Neuroblastoma (NB) is one of the most common solid tumors occurring in children, accounting for 15% of pediatric tumor-related deaths [1]. The clinical courses of NB are very heterogeneous., from fatal progression to spontaneous differentiation and regression, however, the underlying molecular mechanisms remain highly obscure [2]. Despite the use of comprehensive treatment for decades, long-term survival in high-risk patients is lower than 50% [3]. However, relapse is still a frequent cause of treatment failure and approximately 75–80% of single chemotherapy recipients will relapse within one year [4]. Thus, a better understanding of the predictors of recurrence is essential to realize the most effective individualized treatment and improve the survival rate in NB.

Neurotrophins is a prognostic marker for NB development, which are structurally and functionally related growth factors including Nerve Growth Factor (NGF), Brain-derived Neurotrophic Factor (BDNF), NT-3, and NT-4/5, which stimulate the survival, differentiation, and function in neural cells via selective activation of tyrosine kinase receptors (TrkA, TrkB, and TrkC) [5]. BDNF is an alkaline protein which is present in the body as a dimer, mainly in the central nervous system [6]. In addition, BDNF was also detected in peripheral tissues such as heart, lung, and muscle [7]. It is required for the development and maintenance of peripheral sympathetic and sensory neurons, apart from this crucial role, BDNF is involved in oncogenesis [8]. Elevated expression of BDNF has been in several kinds of human malignancies such as prostate cancer, cervical cancer and brain neoplasms, in association with tumor invasion and unresponsiveness to conventional chemotherapeutic regimens^[[9][10][11]]^.

In NB, BDNF/TrkB highly expressed in patients with unfavorable prognosis, and BDNF/TrkB induced NB cell proliferation and survival, while inducing resistance to chemotherapy [12]. Binding of BDNF to TrkB induced homodimerization [13]. Tyrosine residues within the cytoplasm are rapidly phosphorylated and tyrosine kinase regions within the intracellular segment are activated. Then, the reactions activated intracellular PI3K/Akt and Ras/ERK signaling pathways, where the former can inhibit the activity of apoptotic proteins, and the latter can activate anti-apoptotic proteins, thus accelerating tumor growth [14]. Furthermore, the protective effects of BDNF depends on both its concentration and expression [15]. Recently, we have shown that TrkB expression was increased by about nearly 100% in stage 3 and 4 NB [16]. However, BDNF and its receptor TrkB were disproportionately expressed in stage 4 tumors and slight survival effects have been previously reported for exogenously-administered BDNF in the BDNF secreting lines SMS-KCN, SY5Y [17]. However, the distribution and relationship with prognosis of BDNF in NB is hitherto unreported.

In this study, we sought to compare the serum concentration of BDNF between normal people in different postnatal periods and NB children to explore the relationship between BDNF and the occurrence and natural regression of NB. We further investigated the correlation between changes of serum BDNF concentration with clinical features and prognosis in children with NB. We hypothesized that BDNF may contribute to the predictor of NB recurrence.

## Materials and methods

In this trial, we investigated a predictor for relapse of childhood neuroblastoma- BDNF. This prospective clinical trial was initiated with Pediatric Hematology/Oncology Department, Children’s Medical Center of Sun Yet-sen Memorial Hospital and results were reviewed by pediatric blood laboratory technicians. Patients were required to provide written informed consent, including the possible risks of venous blood drawing. This study was divided into two stages, the first stage was NB children and normal people of different ages were included to explore the trend of serum BDNF concentration during human growth and development. In the second stage, 21 NB children were enrolled, and the relationship between the change of serum BDNF concentration and NB prognosis was detected before treatment, during therapy and 2 years after therapy.

### Subjects

#### Phase 1

Phase 1 study was a multi-group sample control trial, where allocation of full-term neonatal, school-age children, healthy adults, and NB children into four groups was conducted at our department over a period of 3 months from March 2017 till May 2017. They were classified into 4 groups: Group 1 included 34 neonates which were full term > 37 completed weeks of gestation, appropriate for gestational age. The umbilical cord blood of newborns with congenital malformations, chromosomal abnormalities, congenital heart disease were excluded from the study. Since the nervous system of children develops faster than that of adults, and there are certain differences in BDNF levels, we divided normal people into two groups, children and adults. Group 2 and 3 were 28 school-age children and 19 healthy adults, which were healthy persons who underwent medical examinations. Children were aged from 3 to 7 and adults were aged from 18 to 35. Group 4 included 6 patients with histologically-proven NB at diagnosis with sufficient tumor tissue and complete follow-up. All diagnoses of tumors were confirmed by histological assessment.

#### Phase 2

Phase 2 study included 21 children with stage 4 NB confirmed by histological assessment from May 2017 till September 2021. The distinction of tumor staging was based on the International Neuroblastoma Staging System (INSS) [15]. The clinical parameters, including pathologies, primary tumor sites, tumor markers, and treatment of NB patients were collected for predictive analysis. All patients were involved in a clinical trial (ChiCTR1800014748) on arsenic trioxide (ATO) combined with conventional chemotherapy for stage 4/M NB [18]. Blood samples were collected at the initial onset, after treatment, and one year after therapy for a control study.

The study was approved by the Ethical Committee of Sun Yat-sen Memorial Hospital of Sun Yat-sen University. Informed verbal consent was obtained from the parents before enrollment of patients.

### Sample collection, processing, and storage

In total, 34 cord blood samples were collected. Blood samples from each subject were collected from the umbilical cord of infants and were then sent to the laboratory for serum separation. Samples were centrifuged at 1500 g for 15 min at room temperature to obtain serum. Hemolytic samples were excluded from analysis. Sera were stored at − 80℃. Then we collected serum samples from healthy children (n = 28) and adults (n = 19). The patients included 21 patients with stage 4 NB in compliance with the diagnostic criteria of the INSS. All serum samples were immediately snap-frozen and stored at − 80℃ until analysis.

### Measurement of BDNF levels in cord blood and plasma

Under the instructions of manufacturer, ELISA kits(Cat. # ELH-BDNF;RayBio) were employed for measuring expression of cord blood BDNF levels. The least measurable dose was determined to be 80 pg/mL, while the intra-assay coefficient of variation was < 10%, and the inter-assay coefficient of variation was < 12%.

Plasma levels of BDNF in the samples were detected through ELISA technique using Quantikine human BDNF immunoassay kit supplied by R&D systems, 614 McKinley Place NE, Minneapolis, MN 55,413, United States of America (R&D Systems, 2006). And this assay employs the quantitative sandwich enzyme immunoassay technique. Briefly, A monoclonal antibody specific for BDNF has been precoated onto the microplate. Standards and samples were added into appropriate wells and any BDNF present was bound to the immobilized antibody. An antibody specific for BDNF was pipetted into the wells. Following washing to remove unbound antibody enzyme, a color reagent was added.The color development is stopped and the absorbance is read.

### Statistical analysis

In the first stage, a descriptive analysis of the baseline characteristics and BDNF levels of all subjects. The adoption of normal distribution of Shapiro—Wilk test. Continuous data were reported as mean ± standard deviation or median (interquartile range). Categorical data were presented as percentage (%). The median statistical description index was P25, P75. Kruskal Wallis H test for K independent samples and Mann-Whitney U test for p2-comparison between multiple groups of samples were conducted, and the test level was set at 0.05. Statistical analyses were performed using SPSS version 21.0 software.

In the second stage, we also studied the influence of pathologies, maximum diameter of tumors, serum NSE, MYCN amplification, and treatment modality on the serum concentrations of BDNF in our population of NB patients using T-test and Pearson’s chi-squared test, respectively. Odds ratio(OR) between remission and relapsed groups was used Logistic regression analysis. Fisher’s statistical method was used to compare the changes of BDNF concentration before and after treatment. A two-sided P value of 0.05 was considered statistically significant. Objectives concerning survival extension (DFS and OS) will be evaluated using Kaplan-Meier’s method. Statistical analyses were performed using SPSS version 21.0 software.

## Results

### Serum concentrations of BDNF at different stages

**Fig. 1:**
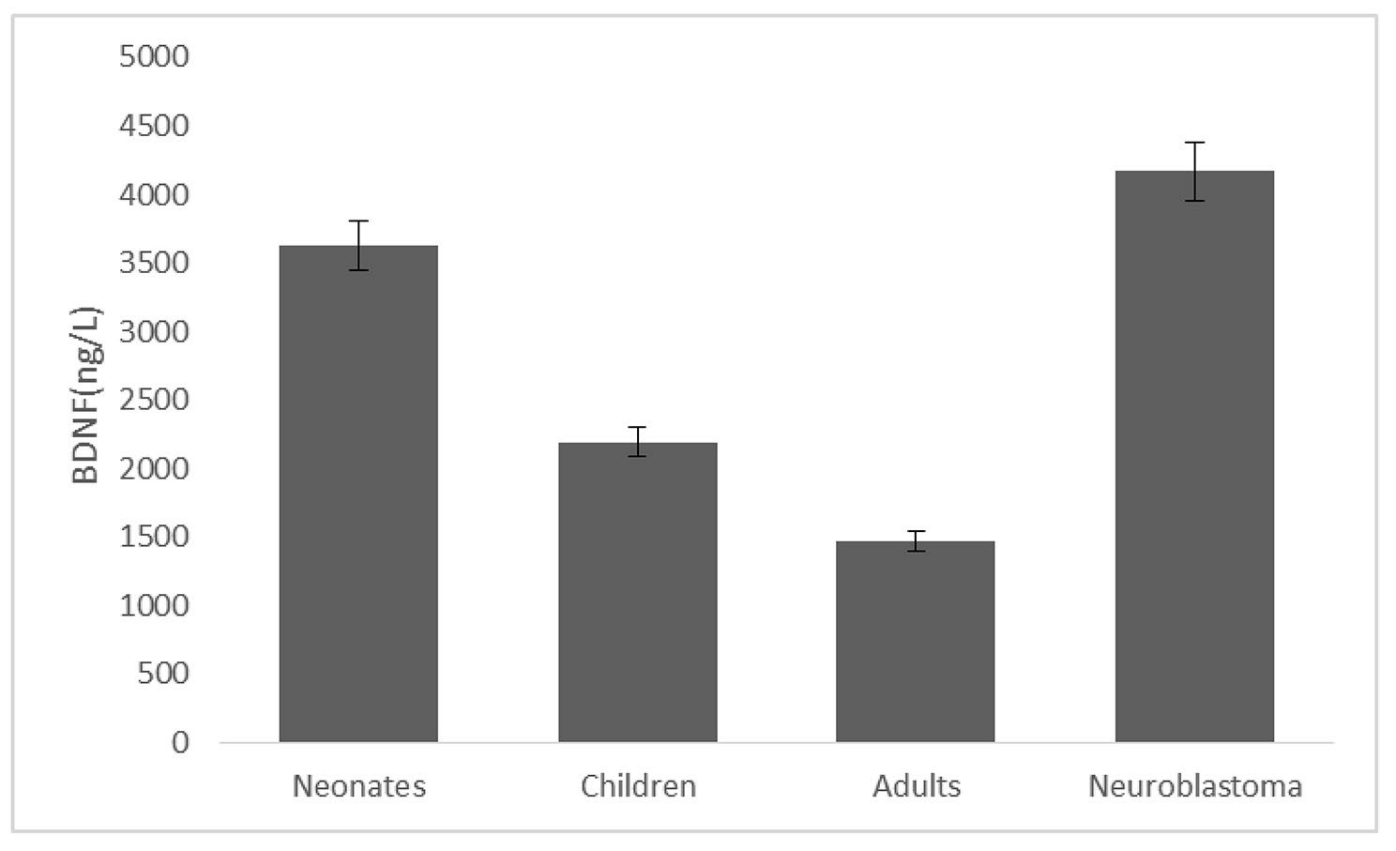
Serum concentration of BDNF in each group (ng/L). (1) The serum concentration of BDNF in NB children group(3–9 years old) and neonatal group (umbilical cord blood at birth) was higher than that in the adult group (16–35 years old) and normal children group (3–7 years old). (2) There was no significant difference in serum concentration of BDNF between the NB children group and newborn group, as well as between the normal children group and adult group

A total of 87 subjects were enrolled in the study, Group 1 of 34 neonates, 16 were males and 18 were females; Group 2 included 16 male and 12 female children, aged 3–7 years with a median age of 5 years; Group 3 of 19 adults, 16 were males and 3 were females, aged 16–35 years, with a median age of 24 years; Group 4 including 6 stage 4 NB patients, 3 were males and 3 were females, aged 3-9years with a median age of 5 years. A normality test was performed before statistical analysis. Thus, we came to a conclusion that serum concentrations in each group obeyed non-normal distribution. Serum concentration levels in each group were as follows. The neonate group: 3628.50 (1755.75, 6449.25) ng/L. The children group: 2195.00 (846.45, 3690.25) ng/L. The adult group: 1471.00 (1132.00, 2056.00) ng/L. The patient group: 4168.00 (2904.25, 5701.25) ng/L. The differences in serum concentrations of neurotrophins between the groups were as follows: (1) The serum concentration of BDNF in the patients and neonates was higher than that of the adults and children. (2) There was no significant difference in BDNF serum concentration between the patient group and the neonatal group, as well as between the children group and the adult group (Fig. 1).

**Table:** 

Group	BDNF (ng/L)	p-value
Neonates	3628.50	0.716
Children	2195.00	0.026
Adults	1471.00	0.006
Neuroblastoma	4168.00	---

## Association of BDNF and relevant clinical features of the patients

Clinical data from the 21 patients with NB enrolled in the observation program are summarized in **Table** 1. All patients had presumed stage 4 NB according to the INSS. The 11 boys and 10 girls were 0.5–13.5 years old at the time of diagnosis, with a median age of 5.1 years. The maximum diameter of the primary tumor was 14–97 mm, with an average of 45 mm. The serum NSE levels in 19 patients (90%) were above the normal value at the initial diagnosis, with a median of 205 nb/dL (range, 18–386 nb/dL). Furthermore, 11 patients had NSE levels of greater than 142 nb/dL. There were 12 patients with bone marrow metastasis, and 5 patients demonstrated MYCN amplification.

**Fig. 2:**
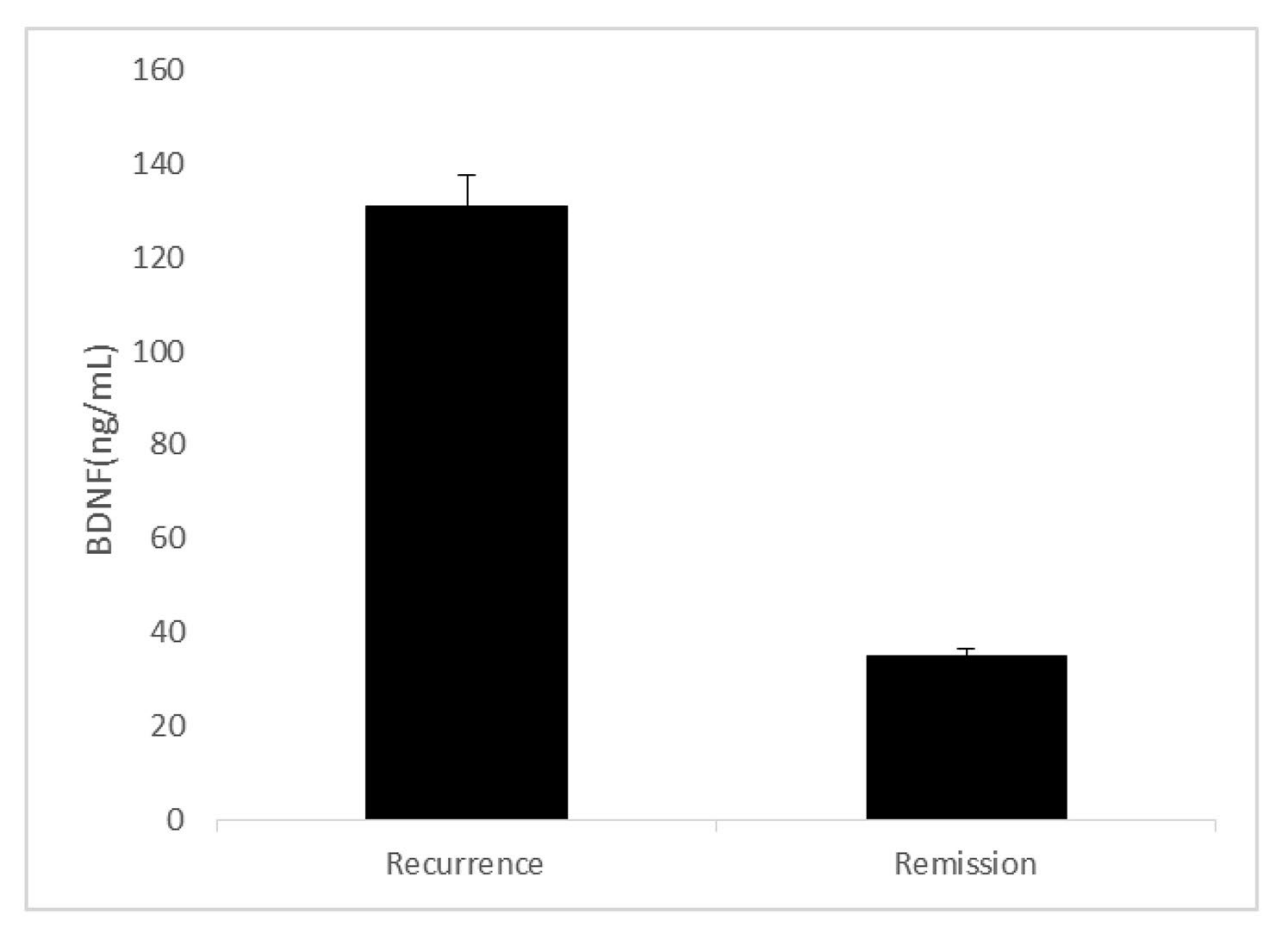
Comparison of BDNF level in children with neuroblastoma with different outcomes. The recurrence group consists of children with recurrence after treatment, the remission group consists of children with remission after treatment (including complete remission and partial remission). The serum concentration of BDNF in the recurrence group was significantly higher than that in the remission group at the beginning, and the difference was statistically significant (P = 0.014)

All 21 children were followed up to 1 year after the end of treatment and divided into recurrence group and remission group according to WHO efficacy evaluation criteria. Complete response was defined as complete disappearance of all lesions for at least 4 weeks. Partial remission was defined as dual-diameter measurable lesions with a maximum diameter reduction of more than 50% for at least 4 weeks. Recurrence was defined as a maximum diameter increase by more than 25% of one or more lesions, or appearance of new lesions. Children in partial and complete remission were assigned to the remission group. According to the current data analysis, there were 9 cases in the recurrence group and 12 cases in the remission group. The serum level of BDNF in the recurrence group was significantly higher than that in the remission group (Fig. 2). The OR between remission and relapsed groups was used Logistic regression analysis: OR = 3.359; 95% CI: 1.297–8.701 (P = 0.014),so serum BDNF were identified as predictors of relapse in neuroblastoma children.

### Disease-free and overall survival

**Fig. 3:**
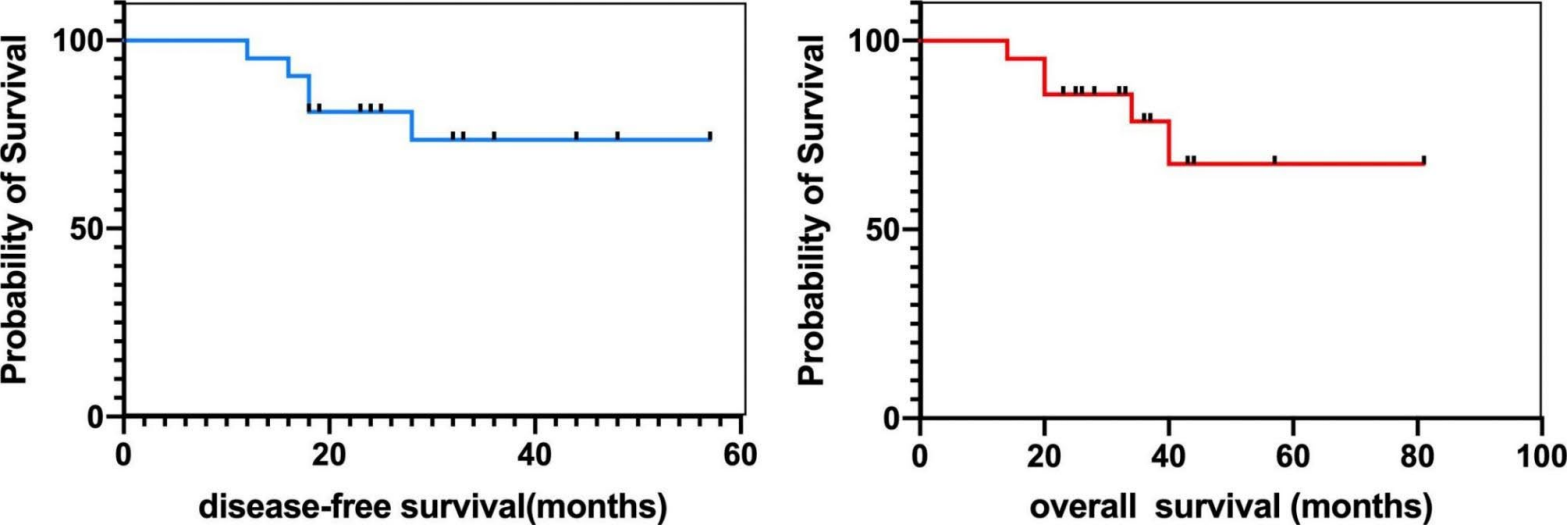
Survival results. Disease-free survival and Overall survival. Vertical marks reflect last follow-up times for censored observations

The median follow-up period was 36.5 months (range 14–81 months). Among 21 patients, 9 patients (42.0%) showed tumor recurrence (Table 1) and 5 children died from neuroblastoma. 76% of alive patients having > 3 years follow-up (Fig. 3). Two relapse children have an intracranial relapse, and these two died soon after treatment failure. The other 7 relapse children have a distant relapse, predominantly in the bone (77.8% of relapse patients who have a distant relapse). There are 11 events in the DFS analysis (52%), in addition to 9 relapses, one child developed a second tumor and the other had drug-related neurotoxicity, but both improved after treatment.

**Table 1:** Association of BDNF and the relevant clinical features of patients

	No	BDNF (ng/mL)	p-value
**Pathologies**			
Ganglioneuroblastoma	4(19%)	98.03	0.310
Neuroblastoma	17(81%)	90.22	
**Tumor maximum diameter**			
≤ 50 mm	9(42%)	83.41	0.090
>50 mm	12(58%)	94.93	
**Serum NSE**			
≤142 ng/mL	10(47%)	59.45	0.045
>142 ng/mL	11(53%)	73.61	
**MYCN amplification**			
Normally expressed	16(77%)	95.65	0.120
Overexpressed	5(23%)	101.71	
**Relapse** ^**a**^			
Negative	12(58%)	35.01	0.014
Positive	9(42%)	131.02	

Abbreviation: NSE = neural specific enolase)

^**a**^ Logistic regression analysis: remission and relapsed groups : [OR = 3.359; 95% CI: 1.297–8.701 (P = 0.014)].

### Expression level of BDNF as a highly specific predictor of relapse

**Fig. 4:**
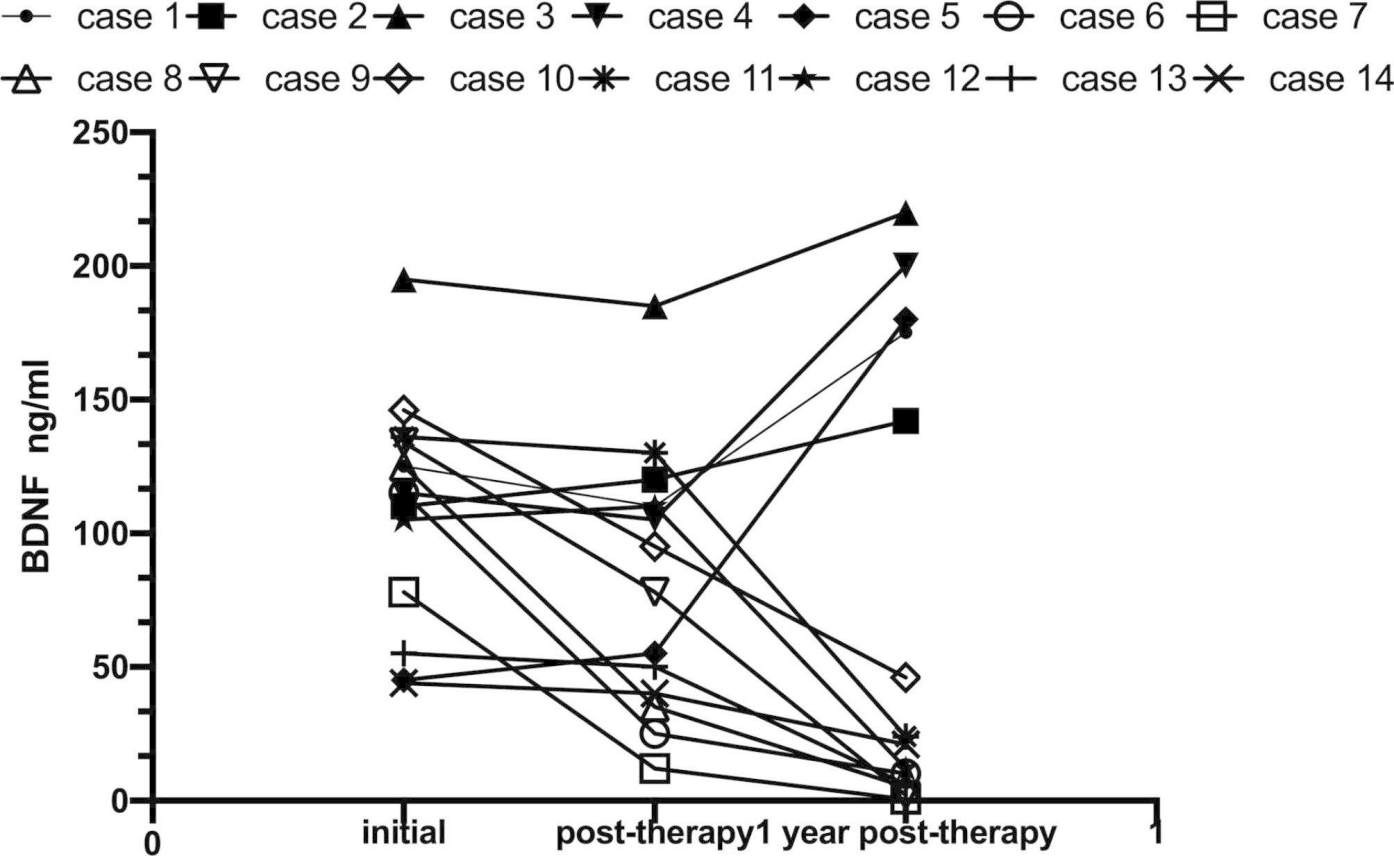
Serum levels of BDNF before and after therapy of patients with NB. Recurrent group (Case 1–5) demonstrated a striking increase of serum BDNF concentration after therapy, while the BDNF concentration of nine children in remission (Case 6–14) decreased significantly

To further investigate the relationship between therapeutic effect and BDNF, 14 children with NB with 1-year post-treatment follow-up were enrolled in this study. Blood samples were collected at the initial onset, after treatment, and one year after therapy. By monitoring serum BDNF values and clinical parameters of these patients, we found that the recurrent group showed serum BDNF concentration was evidently higher after therapy (Case 1–5, Fig. 4). while the BDNF concentration of 9 children in remission decreased significantly after treatment (Case 6–14, Fig. 4). After 1-year long-term follow-up, efficacy was evaluated and changes in BDNF concentration were detected. It was found that five of the six children with elevated BDNF had relapsed (80%) and 1 had complete remission (20%). Due to the small sample size, Fisher’s accurate test was adopted, and the results showed P = 0.005, which implied that the BDNF still did not decrease after treatment, which was an indicator to predict recurrence.

## Discussion

As is well known, NB has a wide spectrum of clinical behavior, which can include spontaneous regression, maturation to a benign ganglioneuroma, or aggressive disease with metastatic dissemination or even death [19]. The characteristics of embryonic origin of NB spontaneous regression attracted many oncologists, where NB was believed to be the most promising to reveal tumor reversal [20]. Neurotrophin is one reason to support several possible mechanisms [21]. TrkB is coexpressed at high levels along with its ligand, BDNF, in clinically and biologically unfavorable tumors, especially those with MYCN amplification [22].

BDNF was the most extensively studied neurotrophin in humans [23]. Further, BDNF was involved in the key processes in the development of the nervous system, including proliferation, migration, differentiation, and synaptogenesis [24]. Some scholars considered that BDNF may produce oncogenic effects by accelerating tumour growth,proliferation and metastasis through various molecular mechanisms and cell-intrinsic pathways [25]. It was hypothesized on the basis of several studies that there must be an steady-state level of BDNF in plasma that ensures the prevention of initiation of cancer [26]. Before our study, BDNF had been shown to play a role in cancer proliferation, invasion and metastasis in central nervous system, due to promote angiogenesis by increasing VEGFR expression through HIF-1α [27].Previous reports of TrkB/BDNF signaling playing a chemoattractant role for development when the early transformation from normal neuroblast to neuroblastoma occurs [28]. And in our finding on the change of serum concentration of BDNF at different periods, an interesting phenomenon was reported. The serum concentration of BDNF decreased from birth to childhood in healthy subjects, whereas it was relatively high in children with NB who were also in the childhood phase. It was found that there was a surge of BDNF and TrkB level during neonatal neuron development [29]. After birth, the brain mainly maintains normal activities, so the BDNF level was relatively lower [30]. Our previous study on NB found that TrkB gene with poor prognosis was highly expressed in stage 4 NB cells with poor prognosis, which was similar to the high expression level of TrkB ligand BDNF in the serum of normal people during the neonatal period [16]. However, BDNF and the corresponding TrkB receptor remained at a high level in children with NB. We speculated that the high level of postnatal serum BDNF which was used as the TrkB receptor was the main reason for the non-regression of some NBs in the neonatal period. Therefore, the BDNF/TrkB signaling pathway plays a vital role in the genesis of NB [31].

Meanwhile, our study was the first to investigate the relationship between different clinical characteristics and BDNF concentration in children with NB, and concluded that children with elevated serum BDNF had elevated NSE at the initial onset of disease and were more likely to relapse after treatment. The high level of serum BDNF expression may be an important factor leading to the continuous progression of NB in children, and it is not affected by the serum levels of NGF and NT-3 [32]. The BDNF/TrkB signaling pathway plays an important role in the genesis and development of NB, and has attracted the attention of researchers in non-nervous system tumors [33].The subsequent inclusion of more cases would enable statistical analysis of the correlation between the high level of serum BDNF at initial onset and tumor risk, clinical stage, efficacy, and long-term prognosis, providing a new, effective, and feasible recurrence predictor for children with NB.

In our study, 21 stage 4 neuroblastoma children received allogeneic hematopoietic stem cell transplantation (alloHSCT) and/or immunotherapy following ATO-basic modified chemotherapy [18]. The result show 9 patients (42.0%) relapsed and 5 children died in the median 36.5 months follow-up period. Overall, 76% of alive patients having > 3 years follow-up. Among the article published in CANCER 2022, high-risk neuroblastoma patients with end-induction residual disease commonly receive post-induction therapy in an effort to increase survival by improving the response before autologous stem cell transplantation (ASCT). The outcomes for cohorts 3-year OS was 85% [34]. This result was a little better than those obtained in our study. A Canadian study recruited 140 high-risk neuroblastoma children 3-year EFS and OS were 71% and 86% [35]. According to a report from the Children’s Oncology Group(COG) stage 4 neuroblastoma patients were treated by postconsolidation therapy combined with immunotherapy targeted to GD2. 64 patients were enrolled, the 3-year EFS was 73.7%, and the OS was 86.0%, respectively [36]. Of these literature-reported patients, the value of the survival rate was comparable to compare our results. We believe that ATO combined chemotherapy-HSCT-immunization combination therapy is a promising therapeutic approach for stage 4 neuroblastoma.

So far, refractory and relapsed neuroblastoma remains a major clinical challenge in pediatric oncology [37].At present, it has been recognized that TrkB was related to the prognosis of NB at home and abroad, but there were no studies on the correlation between BDNF and the prognosis of NB. In several types of cancer, increased expression of BDNF has been implicated in the metastasis [38]. For instance,high levels of BDNF are significantly correlated with the poor overall survival of breast cancer patients [39]. Because BDNF knockdown can reduce cell proliferation and growth [39]. Another study was involved that the presence of human BDNF significantly increases the metastatic potential of colon cancer cells [40]. And this migration was due to BDNF mediated upregulation of heme oxygenase-1 (HO-1) and vascular endothelial growth factor (VEGF) [41]. The results of our study suggest that the high level of serum BDNF expression may be an important factor leading to the continuous progression of NB in children. We further observed the changes of serum BDNF concentration after NB treatment, and the results showed that BDNF increased in children with recurrence but decreased rapidly in children with complete remission. We hypothesized that the significant increase of BDNF in the serum of patients with tumors at the primary site of NB was mainly caused by increased autocrine function of BDNF by NB cells in order to maintain their own proliferation [42, 43]. Although the number of patients was relatively small, our study represents the largest of its kind, using BDNF as a marker of recurrence. It is worthy of further study, and no relevant report has been found at present.

In conclusion, our study has identified an additional role for BDNF in NB tumorigenesis as a potential predictive value for prognosis. Our further studies will be focused on why BDNF concentration does not continuously decrease in NB.

## Data Availability

The datasets used and/or analysed during the current study are available from the corresponding author on reasonable request
